# Is Political Football (PF) Really Just a Harmless Kick-About?

**DOI:** 10.1192/bjo.2026.11768

**Published:** 2026-06-30

**Authors:** David Brunskill

**Affiliations:** Te Ara Kaupare Youth Forensic Service - Midlands, Hamilton, New Zealand

## Abstract

**Aims:**

Enhance PF understanding.

**Methods:**

To ascertain what PF is, when/how/why played, and the potential effects of a ‘game’, a composite definition was developed:

*An ostensibly non-partisan issue or problem that politicians from different parties argue about, primarily in order to obtain advantage for themselves rather than seeking to resolve the issue per se, as to do so may entail disadvantage in terms of acceptance of blame/responsibility, or inadvertently reveal the true scale of the problem, and the resources needed to meaningfully address the same.*

The (sporting) metaphor was extended.An established PF was selected for a worked example - youth offending (YO) in New Zealand (NZ) - in order to explore if an additional underlying psychological process could be identified.

**Results:**

PF should matter to psychiatry.Mental health is itself sometimes designated as a PF, and a psychological lens can help to dissect a phenomenon with potential to affect us all.

Whilst PF is at heart a calculated opportunity for political gain, it also appears to represent the psychological defenses employed by the player, with evidence of a projection-driven othering noted with respect to the worked example (given overwhelming evidence of almost ubiquitous childhood adversity *prior* to YO, it is hard to conceive that the governmental introduction of a ‘boot-camp’ for YO, can be anything other than a deliberate attempt to obscure confronting aspects of societal reality in favour of short-term political gain).

Unsurprisingly, media influences the perceived ripeness of an issue for PF selection.When a PF becomes perennial, it may intersect with other PF issues, and can even appear bigger than it actually is, like a super-moon (NZ YO decreased, 2014-2024).

Reducing a complex issue to binary terms, is also misleading.With respect to YO in NZ, PF robbed the general public of the chance to develop a view on what can be reasonably expected to work in a complex situation i.e. the populist proposal for a ‘boot-camp’ likely won ‘tough-on-crime’ votes, but without reference to the confronting evidence of societal drivers, risks being construed as disingenuous.

**Conclusion:**

Blunt dissection can expose PF for what it actually is (shallow, self-interested psychologically insulated) and what it is not (meaningful, thoughtful, harmless). Thus PF stands as a - very human - obstacle to positive societal change. Better understanding of PF is therefore important, including with respect to the psyche of individuals playing for power.

